# The Impact of Consumers’ Loneliness and Boredom on Purchase Intention in Live Commerce During COVID-19: Telepresence as a Mediator

**DOI:** 10.3389/fpsyg.2022.919928

**Published:** 2022-06-23

**Authors:** Chen Peng, Zhikun Liu, Jong-Yoon Lee, Shanshan Liu, Fang Wen

**Affiliations:** ^1^School of Communication, Linyi University, Linyi, China; ^2^School of Art, Sangmyung University, Cheonan, South Korea; ^3^School of Communication, Nanyang Institute of Technology, Nanyang, China; ^4^School of Design, Sangmyung University, Cheonan, South Korea

**Keywords:** loneliness, boredom, telepresence, influencer-brand image congruence, purchase intention, live commerce

## Abstract

This paper examines the relationship between consumer loneliness, boredom, telepresence, influencer-brand image congruence and purchase intention by investigating consumers of live commerce during the COVID-19 period. With the help of an online survey website, survey data was gathered on 550 Chinese customers who experienced live commerce shopping in China. Although previous studies have shown that consumer boredom and loneliness have an impact on purchase intention, the mechanism of influence remains unclear. As a result, additional research is needed to study the link between boredom and loneliness and customer purchase intention. Consumers’ purchase intention was influenced by their feelings of loneliness and boredom. Telepresence played a mediating role in the impact of loneliness and boredom on purchase intention. Influencer-brand image congruence played a moderating role in the impact of consumers’ boredom on purchase intention. The study results contribute to the research of factors impacting consumers’ purchase intention. In addition, this study can help live commerce merchants better understand the impact factors of consumers’ purchase intention and contribute to the development of live commerce.

## Introduction

Live commerce has been booming in China in recent years and has been welcomed by the market as a new type of shopping (Andronie et al., 2021; Liu et al., 2022). With the attributes of social business and the unique live streaming attributes, live commerce is rapidly improving its position in the consumer market. According to data, China’s live commerce market size exceeds RMB 1.2 trillion in 2020, with an annual growth rate of 197%, and is expected to exceed RMB 4.9 trillion in 2023. Live commerce is rapidly taking over China’s consumer market (iresearch, 2021). Meanwhile, on March 11, 2020, the World Health Organization (WHO) announced the emergence of the COVID-19 outbreak, which has now affected more than 223 countries and territories (Priyadarshini et al., 2020). Studies have shown that COVID-19 can cause physical and mental health damage (Moreno et al., 2020; Xiang et al., 2020; Meda et al., 2021). Preliminary research suggests that the pandemic’s mental challenge will continue to impact society and people for some time (Ahmed et al., 2020; Brenner, 2020). And because people fear that COVID-19 will threaten their lives, they reduce social activities to protect themselves and spend a lot more time at home, resulting in issues like feelings of loneliness and boredom (Brooks et al., 2020). Studies have shown that consumer emotions are closely related to purchase intention (Ma and Wang, 2021; Watson and Popescu, 2021; Zhou et al., 2021; Kliestik et al., 2022). When people feel negative psychological emotions, they buy products to relieve negative emotions and escape from reality (Hoffner and Lee, 2015; Gong et al., 2022). It is worth noting that people would alleviate their loneliness and boredom by purchasing through live commerce (Snyder and Newman, 2019). However, the literature on live commerce research during COVID-19 is insufficient. The mechanisms by which consumers’ feelings of loneliness and boredom impact purchase intention remain unclear. Therefore, there is a need for further research on the mechanisms of how loneliness and boredom generated by consumers during the COVID-19 period affect purchase intention.

**FIGURE 1:**
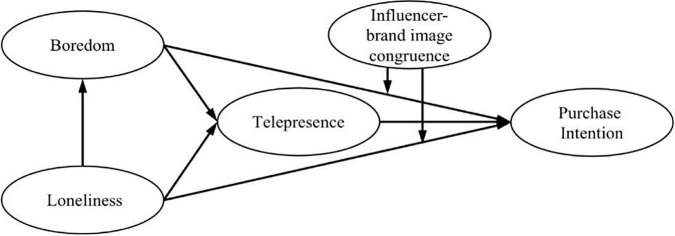
Research model.

This study contributes to the body of knowledge about the factors that influence the purchasing intentions of online shoppers. First, this study enriches the research in related fields by illustrating how consumers’ loneliness and boredom impact purchase intention during the COVID-19 period based on media compensation theory. Specifically, the effects of loneliness, boredom, and telepresence on purchase intention are identified. The study explored the mediating role of telepresence between boredom and purchase intention and loneliness and purchase intention. Second, our study reveals that influencer-brand image congruence moderates the effects of loneliness and boredom on purchase intention. The results show that influencer-brand image congruence enhances the positive impact of loneliness and boredom on purchase intention. Our study also provides practical suggestions for live commerce platforms and merchants to promote live commerce’s sustainable and healthy development. Therefore, this work is crucial since it will provide new information for future consumer psychology and live commerce during the COVID-19 pandemic. Figure 1 shows the research model established in this paper.

## Literature Review and Hypothesis Development

### The Impact of Loneliness on Boredom

The COVID-19 pandemic has made it more difficult for individuals to go outside due to fears of infection, death and other threats from the outbreak. And, as time spent at home increases dramatically, people reduce social and recreational activities, increasing consumer isolation (Bu et al., 2020; Elran-Barak and Mozeikov, 2020; Ausín et al., 2021). When people experience emotions of loneliness, they generally wish to take action to interact *via* online media to compensate for real social interaction (Smith et al., 2021). Therefore, when people feel lonely during the pandemic, they reduce their loneliness by using smartphones and other methods of entertainment to relieve their negative inner feelings (Kardefelt-Winther, 2014; Killgore et al., 2020a). As an essential factor affecting people’s mental health, loneliness is closely related to negative emotions such as boredom and anxiety (Beutel et al., 2017). Some studies show that loneliness leads to higher levels of boredom (Chen, 2020; Li et al., 2021). In the COVID-19 period, boredom increases when people feel lonely at home due to the inability to satisfy their inner social needs. Based on this, the following hypothesis is proposed:

**H1:** Loneliness during the COVID-19 period has an impact on boredom.

### The Impact of Loneliness on Purchase Intention

Loneliness is a subjective state of social isolation, a painful experience of the individual’s lack of contact with others (Russell et al., 1978). Loneliness is often described as a negative emotion associated with isolation and dissatisfaction (Rokach, 2004). As a negative emotion, loneliness can make people feel unhappy and even lead to illnesses such as depression because loneliness can make people’s perceived social status and socialization inconsistent with reality (Russell, 1996). With the rise of loneliness, people often want to take steps to alleviate this negative feeling and, if necessary, even need intervention therapy (Park et al., 2015; Rajendran and Arun, 2020; Yang et al., 2021). Loneliness increases during COVID-19 pandemic when people cannot engage inadequate social and recreational activities due to social distance restrictions and other reasons (Killgore et al., 2020b; Li and Wang, 2020). In this case, people will alleviate their loneliness by purchasing goods. When people feel lonely, they increase their purchase intention and use shopping consumption as a coping means to relieve loneliness (Mandel et al., 2017; Yang et al., 2021). Therefore, this study hypothesized that:

**H2:** Loneliness during the COVID-19 period can have an impact on purchase intention.

### The Impact of Boredom on Purchase Intention

Boredom can be seen as a state of difficulty in concentrating due to constant fatigue while awake (Mikulas and Vodanovich, 1993). COVID-19 causes many people to stay at home for their protection, making people safer and objectively limiting their social activities. People staying at home every day can reduce individual arousal levels and create boredom (van Tilburg and Igou, 2017). People’s reduced social activities and social isolation can increase boredom and reduce people’s life satisfaction (Chao et al., 2020). Studies have shown that boredom is closely related to shopping behavior, and people’s intention and behaviors increase when they feel bored (Lidholm et al., 2017; Bae et al., 2019; Bozaci, 2020; Pelegrín-Borondo et al., 2020). As a result of the common idea that going shopping may both ease boredom and put importance into otherwise mundane events (Sundström et al., 2019). Therefore, to compensate for the lack of social interaction in real life, people increase their purchase intention and even develop an addiction to excessive shopping (El Hedhli et al., 2016; Lidholm et al., 2017; Vinerean et al., 2022). Therefore, this study hypothesized that:

**H3:** Consumer boredom in the COVID-19 period can have an impact on purchase intention.

### The Mediating Effect of Telepresence

Telepresence is an immersive online experience that is a combination of imagined accessibility and immersion, in which consumers are very focused and engaged (Novak et al., 2000; Hopkins et al., 2004; Hyun and O’Keefe, 2012; Beuckels and Hudders, 2016; Cowan and Ketron, 2019). In telepresence, the individual is unaware that they are in a virtual environment created by media such as television or computers at the time (Coyle and Thorson, 2001; Faiola et al., 2013). Telepresence is a standard variable for studying consumer behavior in online environments (Algharabat, 2018). People increase their use of media to compensate for social deficits such as loneliness, boredom, and anxiety (Hollenbaugh and Ferris, 2014; Lim and Kim, 2017). In this process, individuals who feel more lonely may watch media programs and do online shopping to relieve their inner loneliness (Lather and Moyer-Guse, 2011; Minnebo et al., 2014). The emergence of television shopping programs, in particular, has addressed the entertainment and shopping demands of customers (Fritchie and Johnson, 2003). Shopping on television allows consumers to become immersed in the show and engage with the host and other shoppers (Gudelunas, 2002). Consumers can alleviate feelings of loneliness and boredom when shopping on television since they are able to forget about the real world around them (Mollen and Wilson, 2010; Bellman et al., 2014). Through communication and interaction with the hosts of TV shopping programs, individuals can reduce boredom and have a positive psychological experience (Chory-Assad and Yanen, 2005; Derrick et al., 2009; Lim and Kim, 2011). Interacting and shopping with TV hosts help individuals reduce negative emotions, and consumers become immersed in the shopping environment provided by the seller, creating a sense of telepresence. Thus, feelings of loneliness and boredom enhance the telepresence experience. Telepresence could convince customers that they can completely comprehend the goods, allowing them to boost their enjoyment of the occurrence and thus create higher purchase intention (Gao and Li, 2019; Ongsakul et al., 2020; Ye et al., 2020). During the COVID-19 period, less research has been done on the factors influencing consumer purchase intention in TV buying. Customers can communicate with influencers and others more easily in person than they do on television. Customers who see influencers and other consumers market things are more likely to make a buy themselves (Yu and Kim, 2020; Wang et al., 2021). There are just a few options for customers to connect with the host when buying on TV, such as making phone calls or writing letters rather than using multiple communication channels. But the live commerce environment is real-time communication using cell phones face-to-face, and consumers and participants of other live platforms also form multiple communication fields. Therefore, live commerce is more accessible for consumers to feel telepresence than TV shopping, and the interaction methods are more diverse. Based on the above study, these hypothesis was formulated:

**H4:** Loneliness in the COVID-19 period influences purchase intention in live commerce through telepresence.

**H5:** Boredom in the COVID-19 period has an impact on purchase intention in live commerce through telepresence.

### Moderating Role of Influencer-Brand Congruence

In live commerce, influencers play the role of merchandise advocates in the sales process. Many social media influencers have enormous followings, and their fans trust the things they advocate, which is regarded to be the marketing value of the influencers to the companies in these social media industries (De Veirman et al., 2017; Kemp et al., 2019; Lou and Yuan, 2019). Influencers significantly impact consumers’ shopping decisions as product advocates and opinion leaders (Casaló et al., 2020). In previous studies, spokesperson-brand image congruence is an important influencing factor for consumers’ purchase intention. Product spokespersons represent corporate culture and image and are of great significance in the consumer shopping process (Escalas and Bettman, 2005; Shan et al., 2020). When customers believe the spokesperson’s image matches the product’s image, the product commercial and the brand’s image are more positively received by customers, the research revealed (Akbar, 2019; Lee et al., 2019; Haobin Ye et al., 2021). When consumers feel that the spokesperson’s brand image is inconsistent, it decreases the consumer’s favorability of the product and can reduce their willingness to purchase the product (Paul and Bhakar, 2018; Deska et al., 2022). Although consumers’ feelings of loneliness and boredom can have an impact on purchase intention, when influencer images are less consistent with brand images, it may reduce consumers’ trust in the product and decrease the shopping experience. Consumers are less likely to have purchase intention for products with low influencer-brand image congruence. Spokesperson-brand image congruence strengthens consumers’ willingness to shop, but research exists primarily in television shopping or in social shopping (Skupski, 2019; Arora et al., 2021). Fewer studies have been conducted in the COVID-19 period on the influencers’ brand image congruence in live commerce. Consumers in social media will trust influencers more than traditional celebrities and show more positive attitudes toward products endorsed by influencers (Jin et al., 2018; Kim and Kim, 2021; Li and Peng, 2021; Pop et al., 2022). Whether influencer-brand image congruence enhances the effect of loneliness and boredom on shopping intention is a question worthy of further study. Therefore, this study hypothesized that:

**H6:** Influencer-brand image congruence enhances the positive effect of loneliness on purchase intention in live commerce.

**H7:** Influencer-brand image congruence enhances the positive effect of boredom on purchase intention in live commerce.

## Materials and Methods

### Participants

In order to test the research model and related hypotheses, this paper adopts a questionnaire survey method to collect data. 550 Chinese live commerce consumers participated in this study and completed an informed consent form before completing the questionnaire. Using the WENJUANXING data website, a questionnaire was employed to gather information. It was gathered between February 10, 2022 and February 25, 2022.

### Variable Measurement

#### Loneliness

The measure of loneliness was modified from the revised Loneliness Scale by Tian et al. (2012). The scale consists of 20 items (e.g., “Do you feel isolated?”). The response options on the questionnaire ranged from 1 (strongly disagree) to 5 (strongly agree). Higher scores reflect higher levels of individual loneliness. Cronbach’s α = 0.835.

#### Boredom

The boredom scale revised by Lee and Zelman was used in this study (Lee and Zelman, 2019). The scale contains 12 items (e.g., “Usually, I am less able to find things that interest me”). The scale has shown good reliability and validity in previous studies (Elhai et al., 2018). The response options for the questions ranged from 1 (strongly disagree) to 5 (strongly agree). Higher scores reflect higher levels of individual boredom. In our study, the internal rate of reliability of the questionnaire was high with a Cronbach’s α = 0.867.

#### Telepresence

As revised by Kim and Biocca the telepresence scale was used in this study (Kim and Biocca, 1997). The scale contains nine measures (e.g., when I use live commerce, I forget the reality of my environment). Cronbach a = 0.815.

#### Influencer-Brand Image Congruence

The study was adapted from a scale developed by Haobin Ye et al. (2021). The scale contains 3 measures (e.g., do you think the influencer’s image is consistent with the image of the company). The questionnaire response options range from 1 (strongly disagree) to 5 (strongly agree). Cronbach’s α = 0.884.

#### Purchase Intention

The questionnaire was based on a questionnaire developed by Li et al. (2002). The scale contained four measures (e.g., how likely you think it is to buy the product) (Li et al., 2002). The response options of the questionnaire ranged from 1 (strongly disagree) to 5 (strongly agree). Cronbach’s α为0.759.

### Procedure

The Academic Research Ethics Committee of Linyi University approved the study (No. LYU20220105). The study questionnaire was answered anonymously, and they were instructed on how to complete the survey. Informed consent was obtained from all participants before the study, and participants could withdraw during the response process.

### Data Analyses

Statistical analyses were performed using SPSS 26.0 software. Specifically, first, descriptive statistics and correlation analyses were conducted for the main variables. Second, the effect of loneliness on boredom was analyzed by regression using SPSS. In addition, the mediating and moderating roles of telepresence in loneliness, boredom, and purchase intention were analyzed using model 5 of SPSS PROCESS macro (Preacher and Hayes, 2008). SPSS PROCESS macro can provide several models to perform analysis of processes such as mediation and moderation effects.

## Results

### Descriptive Statistics

In the 550 valid survey samples, there are a total of 273 females and 277 males. Sample loneliness mean = 3.52, skewness = –6.38, kurtosis = –0.855. sample boredom mean = 3.43, skewness = –8.56, kurtosis = –0.534. More than 65.82% are people aged 18–32, 75.09% of the samples have a junior college degree or above. Most people spent an average of less than 1 h on live commerce every day. The descriptive statistics of our survey samples are shown in Table 1.

**TABLE 1:** Descriptive characteristics of the sample (*N* = 550).

Characteristics		Frequency	The percentage
Gender	Male	277	50.36
	Female	273	49.67
Age	18 Years old and below	103	18.73
	18–25 Years old	169	30.73
	26–32 Years old	193	35.09
	33–40 Years old	29	5.27
	40 Years old and above	56	10.18
Education	High school and below	137	24.91
	Junior college degree	208	37.82
	Bachelor’s degree	176	32.00
	Master’s degree or above	29	5.27
Frequency of use (hours)	Less than 0.5	248	45.09
	0.5–1	260	47.28
	1–3	24	4.36
	Above 5	18	3.27

Table 2 summarizes the descriptive statistical results and correlations between the main variables. Correlation analysis showed that loneliness was positively correlated with boredom, telepresence, and the purchase intention process. Boredom was positively correlated with telepresence and purchase intention, and Purchase intention was positively correlated with endorser influencer-image congruence.

**TABLE 2:** Correlations between variables.

Variables	*M*	*SD*	1	2	3	4	5
Loneliness	3.5156	0.96004	1				
Boredom	3.428	0.99126	0.382**	1			
Telepresence	3.4986	1.02513	0.358**	0.321**	1		
PI	3.4232	0.83204	0.290**	0.205**	0.292**	1	
IBC	3.1085	1.32318	0.044	–0.013	0.002	0.103*	1

*p < 0.05; **p < 0.01.

*N = 550. M, mean; SD, standard deviation; PI, Purchase Intention; IBC, Influencer-brand image congruence.*

Table 3 depicts the relationship between consumer loneliness, boredom, and purchase intention during COVID-19. Specifically, loneliness had an impact on boredom (β = 0.382, *SE* = 0.035, *t* = 9.669). In addition, loneliness had an impact on purchase intention (β = 0.183, *SE* = 0.037, *t* = 4.961). Boredom has an impact on purchase intention (β = 0.106, *SE* = 0.036, *t* = 2.964). Hypothesis 1 and Hypothesis 2 and Hypothesis 3 are supported.

**TABLE 3:** Standardized parameter estimates for the direct and indirect effects of the hypothesized model.

Relationship	β	*SE*	95% CI		Results
			*Lower*	*Upper*	
Direct effect					
Loneliness→boredom	0.382	0.035	0.314	0.474	Supported
Loneliness→PI	0.183	0.037	0.111	0.256	Supported
Boredom→PI	0.106	0.036	0.036	0.176	Supported
Indirect effect					
Loneliness→TP→PI	0.067	0.016	0.038	0.100	Supported
Boredom→TP→PI	0.068	0.016	0.040	0.101	Supported

*n = 550, bootstrapping randomly sampled 5,000 times.*

Mediation analysis was performed to test the mediating role of telepresence in the association between loneliness, boredom, and purchase intention. To test the mediating role of escape motivation between loneliness, boredom and purchase intention, we ran the PROCESS macro, model 5, in SPSS with 5,000 bootstrapping samples (Preacher and Hayes, 2008). All data were standardized. Telepresence exhibited a significant mediating effect (Table 3). We suggested that telepresence plays a mediating role between loneliness and purchase intention (β = 0.067, 95% confidence interval of bootstrapping = 5,000 is [0.038, 0.100], excluding 0). Moreover, telepresence exerts a mediating effect on boredom and purchase intention (β = 0.068, 95% confidence interval of bootstrapping = 5,000 is [0.040, 0.101], excluding 0). Thus, telepresence partially mediates the relationship between loneliness, boredom and purchase intention.

**FIGURE 2:**
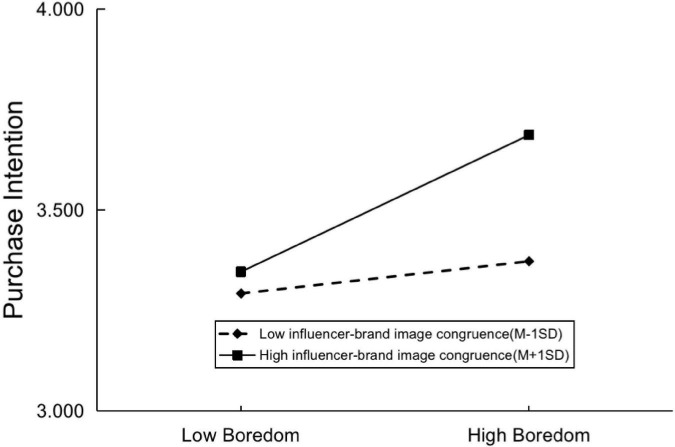
Showing moderation.

To test the moderating roles of influencer-brand congruence, we ran the PROCESS macro (PROCESS Model 5; Preacher and Hayes, 2008). Influencer-brand congruence had a significant moderating effect on the relationship between boredom and purchase intention, results shown in Table 4 (β = 0.049, *t* = 2.024). To illustrate the moderating effect presented above, we conducted a simple slope test. We divided telepresence into high (M + 1 SD) and low (M –1 SD) groups to better explain telepresence in moderating boredom and purchase intention (Figure 2). The results revealed that when the influencer-brand congruence was high (mean + 1 SD), boredom exerted a significant influence on the purchase intention of live commerce consumers (*b* = 0.171, *t* = 3.534, *p* < 0.001). When the influencer-brand congruence was low (mean –1 SD), boredom exerted no significant negative impact on the purchase intention of live commerce consumers (*b* = 0.040, *t* = 0.842, *p* = 0.40). Therefore, We found that influencer-brand congruence exerts a significant moderating effect on boredom and purchase intention (β = 0.049, 95% confidence interval of bootstrapping = 5,000 is [0.072, 0.157], excluding 0). Hence, Hypothesis 4 and Hypothesis 5 and Hypothesis 6 are supported.

**TABLE 4:** Moderation analysis.

Relationship	β	*SE*	95% CI		*t*	Results
			*Lower*	*Upper*		
Loneliness × IBC → PI	0.047	0.025	–0.002	0.096	1.868	Not supported
Boredom × IBC → PI	0.049	0.024	0.001	0.098	2.024	Supported

*PI, Purchase Intention; IBC, Influencer-brand image congruence.*

## Discussion

This study was constructed to explore the potential mechanisms between loneliness, boredom and consumer shopping intention during COVID-19. Telepresence is a potential mediator of loneliness, boredom and shopping intention, while influencer-brand congruence is a potential moderator to explain the influence of loneliness on purchase intention. Loneliness is a factor in boredom for live commerce customers in particular. To put it another way, loneliness has an effect on boredom. Therefore, the study is consistent with the previous findings (Bu et al., 2020; Elran-Barak and Mozeikov, 2020; Killgore et al., 2020a; Ausín et al., 2021; Li et al., 2021). Consumers’ loneliness and boredom positively predicted purchase intention in live commerce. When consumers feel loneliness and boredom, they are motivated to shop through live commerce, consistent with previous research findings (Lidholm et al., 2017; Jiang et al., 2018; Lee and Zelman, 2019; Yang et al., 2021; Vinerean et al., 2022). Telepresence mediates consumer loneliness, boredom and purchase intention (Lim and Kim, 2017; Gao and Li, 2019; Ongsakul et al., 2020). Loneliness and boredom affect consumers’ willingness to shop through telepresence. When consumers are lonely, if telepresence increases, it makes them more likely to have purchase intention. Consumers have reduced their social behavior during the COVID-19 period for various reasons, so they need to compensate for the lack of interpersonal behavior through the Internet and other means. A high influencer-brand image congruence is more likely to make consumers willing to purchase intent, consistent with previous research findings (Ruiz-Equihua et al., 2020; Kim and Kim, 2021; Pop et al., 2022). In other words, when consumers are bored, interesting live commerce can provide a way to eliminate boredom and make consumers feel interesting, which is inseparable from the influencer’s charm. The statistics show that influencer-brand congruence has a moderating effect on the relationship between boredom and purchase intention. However, influencer-brand image congruence did not act as a moderator between loneliness and purchase intention, possibly since, during the pandemic, consumers sought other social outlets to alleviate their loneliness when they felt lonely.

## Implications

When it comes to customers’ purchase intention, previous research has neglected to take into account COVID-19’s results on emotions of loneliness, boredom, telepresence, and influencer-brand congruence. The findings of this study provide important insights for live commerce platforms and merchants. First, given the importance of consumer loneliness and boredom in purchase intention during the COVID-19 period, live commerce platforms and anchors should develop new features that reduce consumer loneliness and boredom to promote consumers’ purchase intention. Second, current research has found that consumers’ telepresence mediates feelings of loneliness, boredom and purchase intention, so live commerce platforms need to improve picture and sound quality to ensure consumers’ telepresence. In addition, influencer-brand image congruence can strengthen the influence of boredom on purchase intention. Especially, retailers using live commerce should focus on enhancing their brand image in order to boost the likelihood that customers will make a purchase during the COVID-19 period. Therefore, when consumers are bored, merchants in live commerce should choose products with high influencer-brand image congruence to sell and promote the sales of products.

## Conclusion

This research sheds light on the psychological underpinnings of consumer purchasing behavior during the COVID-19 period and sheds light on the elements that influenced consumers’ buying intentions. If we want to build the live commerce business, we need to understand the psychology of consumers. This study constructed a moderated mediation model of the relationship between loneliness, boredom, telepresence, influencer-brand congruence and purchase intention during the COVID-19 pandemic and verified the mechanism of loneliness and boredom on the customer purchase intention during the COVID-19 pandemic through empirical methods. Research in the subject of live commerce is enriched by this study, which gives a novel viewpoint on the investigation of customer purchase intention.

Although this study can provide some insight into the factors influencing consumers’ purchase intention, this study has some limitations. First, all data in the study were filled in by consumers themselves, which may lead to bias in consumers’ recall. Second, COVID-19 is still very severe in some areas of China, but the outbreak is under control in other places, which may affect the data’s accuracy. Third, a cross-sectional study cannot determine a causal relationship between loneliness, boredom and purchase intention. This study only examined the regulatory impact of influencer-brand congruence, but it may also be utilized as an antecedent variable to affect purchase intention. Thus future research can further deepen and expand on this. Using a longitudinal survey to evaluate the influence of additional characteristics such as resilience on consumer purchase intention is recommended in future research.

## Data Availability Statement

The original contributions presented in this study are included in the article/supplementary material, further inquiries can be directed to the corresponding author/s.

## Ethics Statement

The studies involving human participants were reviewed and approved by the Academic Research Ethics Committee of Linyi University. The patients/participants provided their written informed consent to participate in this study.

## Author Contributions

All authors listed have made a substantial, direct, and intellectual contribution to the work, and approved it for publication.

## Conflict of Interest

The authors declare that the research was conducted in the absence of any commercial or financial relationships that could be construed as a potential conflict of interest.

## Publisher’s Note

All claims expressed in this article are solely those of the authors and do not necessarily represent those of their affiliated organizations, or those of the publisher, the editors and the reviewers. Any product that may be evaluated in this article, or claim that may be made by its manufacturer, is not guaranteed or endorsed by the publisher.
